# Circ-FOXM1 contributes to cell proliferation, invasion, and glycolysis and represses apoptosis in melanoma by regulating miR-143-3p/FLOT2 axis

**DOI:** 10.1186/s12957-020-01832-9

**Published:** 2020-03-17

**Authors:** Shan Tian, Gangwen Han, Lulu Lu, Xiangyu Meng

**Affiliations:** 1 Dermatology, Peking University International Hospital, Life Park Road No.1 Life Science Park of Zhong Guancun, Chang Ping District, Beijing, 102206 China; 2 Central Laboratory, Peking University International Hospital, Beijing, China

**Keywords:** Melanoma, Circ-FOXM1, miR-143-3p, FLOT2

## Abstract

**Background:**

Numerous literatures have demonstrated that circular RNAs (circRNAs) are involved in multiple types of tumors. However, the effects of circRNAs in melanoma are not very clear. In this study, we aimed to investigate the roles and mechanisms of circ-FOXM1 in melanoma.

**Methods:**

Quantitative real-time polymerase chain reaction (qRT-PCR) was conducted to determine the expression of circ-FOXM1, microRNA-143-3p (miR-143-3p), and Flotillin 2 (FLOT2) mRNA. 3-(4,5-Dimethyl-2-thiazolyl)-2,5-diphenyl-2-H-tetrazolium bromide (MTT) assay, flow cytometry analysis, and transwell assay were employed to test cell proliferation, apoptosis, and invasion, respectively. The glucose consumption and lactate production were examined by specific kits. Western blot assay was utilized for the detection of hexokinase2 (HK2), pyruvate kinase isozyme type M2 (PKM2), and FLOT2. Dual-luciferase reporter assay and RNA immunoprecipitation (RIP) assay were employed to verify the targeting association between miR-143-3p and circ-FOXM1 or FLOT2. A murine xenograft model was established to explore the effect of circ-FOXM1 in vivo.

**Results:**

Circ-FOXM1 was elevated and miR-143-3p was reduced in melanoma tissues and cells. Circ-FOXM1 deficiency impeded cell proliferation, invasion, and glycolysis and facilitated cell apoptosis in melanoma in vitro and tumorigenesis in vivo. Circ-FOXM1 acted as a sponge of miR-143-3p and the impacts of circ-FOXM1 silencing on cell proliferation, apoptosis, invasion, and glycolysis were overturned by miR-143-3p deletion. Moreover, FLOT2 was a target gene of miR-143-3p and FLOT2 overexpression rescued the inhibitory effect of miR-143-3p on melanoma progression.

**Conclusion:**

Circ-FOXM1 facilitated the development of melanoma by upregulating FLOT2 through miR-143-3p.

## Background

Melanoma is one of the most notoriously invasive neoplasia, and it originates from cells that produce melanin [1]. The prognosis of melanoma remains dismal because melanoma is refractory to the current therapies [2, 3]. Moreover, the exact cause of malignant melanoma is very complex and poorly understood. It is therefore essential to explore the molecular pathogenesis of melanoma and develop novel treatment targets for patients with melanoma.

Circular RNAs (circRNAs) are a series of novel identified non-coding RNAs (ncRNAs) with covalently closed-loop structures [4]. With the improvements of bioinformatics analysis and high throughput sequencing, more and more circRNAs have been discovered and identified to participate in the regulation of human tumors [5, 6]. For example, circ-ABCB10 aggravated breast cancer carcinogenesis via promoting the growth of tumor cells [7]. Circ_0020397 was elevated in colorectal cancer (CRC) and facilitated CRC cell viability and motility and impeded apoptosis [8]. Circ-FOXM1 (also termed as circ_0025039) expression has been identified to be raised in non-small lung cancer (NSCLC) and contributed to NSCLC cell progression [9]. These reports indicated that circRNAs played a crucial role in the development of cancers. Nonetheless, only a very small number of circRNAs are identified in melanoma yet and the effects of circ-FOXM1 in melanoma are still not very clear. The purpose of this study is to explore the exact roles and mechanisms of circ-FOXM1 in melanoma.

MicroRNAs (miRNAs), a type of short ncRNAs (~ 22 nucleotides), mainly alter gene expression via recognizing the 3′ untranslated region (3′UTR) of targeted mRNAs at the posttranscriptional level [10]. Diverse miRNAs were proved to function as tumor suppressors to take part in the progression of melanoma. For instance, Li et al. claimed that miR-155 could impede cell proliferation and motility in malignant melanoma by binding to CBL [11]. Zhao et al. declared that miR-140-5p elevation hindered the malignant behaviors of melanoma cells via interacting with SOX4 [12]. Moreover, miR-143-3p was identified to be decreased and acted as an essential regulator in melanoma [13, 14]. Nevertheless, the potential mechanisms of miR-143-3p underlying melanoma are far from being addressed. Flotillin 2 (FLOT2) has been proved to play its tumorigenic role in diverse human cancers, including melanoma [15, 16]. However, whether miR-143-3p can target FLOT2 to participate in the development of melanoma has not been unraveled.

In the presented research, the expression patterns of circ-FOXM1, miR-143-3p, and FLOT2 in melanoma were determined. Furthermore, the roles and regulatory mechanisms of circ-FOXM1 were investigated by function and mechanism analysis.

## Materials and methods

### Tissue collection

After the work was permitted by the Ethics Committee of Peking University International Hospital and written informed consents were provided by the patients, 30 pairs of melanoma tissues and adjacent normal tissues were harvested from melanoma patients through surgery at Peking University International Hospital. The tissue specimens were preserved at − 80 °C before use.

### Cell culture and cell transfection

Normal human epidermal melanocytes (HEMn) and melanoma cells (A2058 and A375) were all bought from the American Type Culture Collection (ATCC, Manassas, VA, USA). These cells were grown in Dulbecco’s modified Eagle’s medium (DMEM; Gibco, Grand Island, NY, USA) containing 10% fetal bovine serum (FBS; Gibco) and 1% penicillin-streptomycin (Gibco) at 37 °C in a humidified incubator with 5% CO_2_.

The overexpression vector of circ-FOXM1 (circ-FOXM1), the overexpression vector of FLOT2 (FLOT2) and their control (pcDNA), small interfering RNA (siRNA) against circ-FOXM1 (si-circ-FOXM1) and negative control (si-NC), mimics of miR-143-3p (miR-143-3p) and control mimic (miR-NC), inhibitors of miR-143-3p (anti-miR-143-3p) and its control (anti-miR-NC), and short hairpin RNA against circ-FOXM1 (sh-circ-FOXM1) and its control (sh-NC) were bought from GeneCopoeia (Guangzhou, China). The synthetic vectors or oligonucleotides were transfected into cells using Lipofectamine 2000 (Invitrogen, Carlsbad, CA, USA).

### Quantitative real-time polymerase chain reaction (qRT-PCR)

Following total RNA was extracted from tissues and cells with TRIzol reagent (Invitrogen), the RNAs were reversely transcribed into cDNAs with PrimeScript™ RT reagent Kit (Takara, Dalian, China) or All-in-One™ miRNA qRT-PCR Detection Kit (GeneCopoeia). Then qRT-PCR was carried out on an ABI 7500 PCR system (Applied Biosystems, Foster City, CA, USA) using SYBR Premix Ex Taq II (Takara). The relative expression was measured using the 2^-ΔΔCt^ method. Glyceraldehyde 3-phosphate dehydrogenase (GAPDH) or U6 was utilized as an internal control. The primers were circFOXM1: 5′-TTCCCTGCACGACATGTTTG-3′ and R: 5′-CTCTCAGTGCTGTTGATGGC-3′); miR-143-3p: (F: 5′-GGGGTGAGATGAAGCACTG-3′ and R: 5′-CAGTGCGTGTCGTGGAGT-3′); FLOT2: (F: 5′-GGCAGTAGGAAACTGAGGAAGCT-3′ and R: 5′-GGACTGGTCTTCCCAGCCCTAAA-3′); GAPDH: (F: 5′-ATGGGGAAGGTGAAGGTCG-3′ and R: 5′-GGGGTCATTGATGGCAACAATA-3′); and U6: (F: F: 5′-CTCGCTTCGGCAGCACATATACTA-3′ and R: 5′-ACGAATTTGCGTGTCATCCTTGCG-3′).

### 3-(4,5-Dimethyl-2-thiazolyl)-2,5-diphenyl-2-H-tetrazolium bromide (MTT) assay

For the detection of cell proliferation, cells were seeded onto 96-well plates (Corning Incorporated, Corning, NY, USA) after relevant transfection. Then 20 μL MTT (5 mg/mL; Sangon, Shanghai, China) was added to each well at 24 h, 48 h, and 72 h and kept for an additional 4 h. Afterward, the formazan crystals were dissolved using 150 μL dimethyl sulfoxide (DMSO; Sangon). The optical density was tested at 490 nm with a microplate reader (Bio-Rad Laboratories, Hercules, CA, USA).

### Flow cytometry analysis

The apoptosis of A2058 and A375 cells was evaluated through Annexin V-fluorescein isothiocyanate (FITC)/propidium iodide (PI) Apoptosis Detection Kit (Vazyme, Nanjing, China). In brief, A2058 and A375 cells were harvested, washed, and resuspended in binding buffer following relevant transfection. Then cells were kept for 15 min with 5 μL AnnexinV-FITC and 10 μL PI in the dark. The rate of apoptosis was analyzed with a flow cytometry (BD Biosciences, San Jose, CA, USA) within 1 h.

### Transwell assay

Transwell insert chambers (Corning Incorporated) coated with Matrigel (Solarbio Beijing, China) was employed for the analysis of cell invasion capacity. Briefly, A2058 and A375 cells (5 × 10^5^ cells/well) were digested in DMEM (Gibco) and seeded in the top chamber. DMEM (Gibco) including 10% FBS (Gibco) was added to the bottom chamber. Twenty-four hours later, cells that are still on the upper chamber were removed and cells that invaded to the lower chamber were fixed with paraformaldehyde (Sangon), stained with crystal violet (Solarbio), and then estimated under a microscope (Olympus, Tokyo, Japan).

### Glucose consumption and lactate production assays

After relevant transfection, A2058 and A375 cells were seeded in 6-well plates for 12 h. The levels of glucose consumption and lactate production were examined using Glucose Assay Kit (Sigma-Aldrich, St. Louis, MO, USA) and Lactate Assay Kit (Sigma-Aldrich) based on the manufacturer’s instructions.

### Western blot assay

Total protein in tissues and cells was extracted using RIPA buffer (Beyotime, Shanghai, China) and determined using a BCA Protein Quantification Kit (Vazyme). Twenty micrograms of proteins were separated by 10% sodium dodecyl sulfonate-polyacrylamide gel (SDS-PAGE; Solarbio). Then the protein samples were transferred onto polyvinylidene difluoride membranes (Pall Corporation, New York, NYC, USA). Thereafter, the membranes were blocked with non-fat milk for 1 h and probed with primary antibody: hexokinase2 (HK2; ab209847; Abcam, Cambridge, MA, USA), pyruvate kinase isozyme type M2 (PKM2; ab137852; Abcam), FLOT2 (ab181981; Abcam), or GAPDH (ab9485; Abcam) overnight at 4 °C. After incubation with secondary antibody (ab205719; Abcam) for 2 h at room temperature, the protein bands were examined by an enhanced chemiluminescence reagent (Vazyme).

### Dual-luciferase reporter assay

The fragments of circ-FOXM1 and FLOT2 containing the predicted wild-type or mutant complementary sequences of miR-143-3p were cloned into pmirGLO plasmids (Promega, Madison, WI, USA), named as WT-circ-FOXM1, MUT-circ-FOXM1, FLOT2 3′UTR-WT, and FLOT2 3′UTR-MUT, respectively. Then cells were cultured in 24-well plates and miR-143-3p or miR-NC together with indicated luciferase reporter vector were co-transfected into A2058 and A375 cells. Forty-eight hours later, Dual-Luciferase Reporter Assay Kit (Promega) was adopted to detect the luciferase activity.

### RNA immunoprecipitation (RIP) assay

The EZ-Magna RIP kit (Millipore) was utilized for RIP assay. Firstly, A2058 and A375 cells were lysed in RIP lysis buffer. Then, cell lysates were incubated with magnetic beads coated with antibody against Argonaute2 (Ago2; Abcam) or immunoglobulin G (IgG; Abcam) for 6 h. Finally, the RNAs in the magnetic bead-binding complexes were purified and subjected to qRT-PCR assay.

### Murine xenograft model

BALB/c nude mice were bought from Shanghai SLAC Laboratory Animals Co., Ltd. (4–6 weeks old; Shanghai, China) and assigned to 2 groups (7 mice/group). Sh-circ-FOXM1 or sh-NC was transfected into A375 cells and then the cells were subcutaneously injected into the right side of the back of the mice. Eight days later, tumor length (*L*) and width (*W*) were monitored every 4 days. Tumor volume was calculated by (*L* × *W*^2^)/2. Following 28 days of injection, the mice were euthanized by cervical dislocation and the tumors were collected, weighted, and saved at − 80 °C for further experiments. The steps were permitted by the Ethics Committee of Animal Research of Peking University International Hospital.

### Statistical analysis

All experiments were repeated three times. The data were exhibited as mean ± standard deviation (SD) and processed using software GraphPad Prism 7 (GraphPad Inc., La Jolla, CA, USA). The difference was estimated by Student’s *t* test or one-way analysis of variance (ANOVA). Spearman’s correlation coefficient analysis was performed to analyze the correlation between levels of miR-143-3p and circ-FOXM1 or FLOT2 in melanoma tissues. It was regarded as statistically significant if *P* value was less than 0.05.

## Results

### Circ-FOXM1 was increased and miR-143-3p was decreased in melanoma tissues and cells

**Fig. 1:**
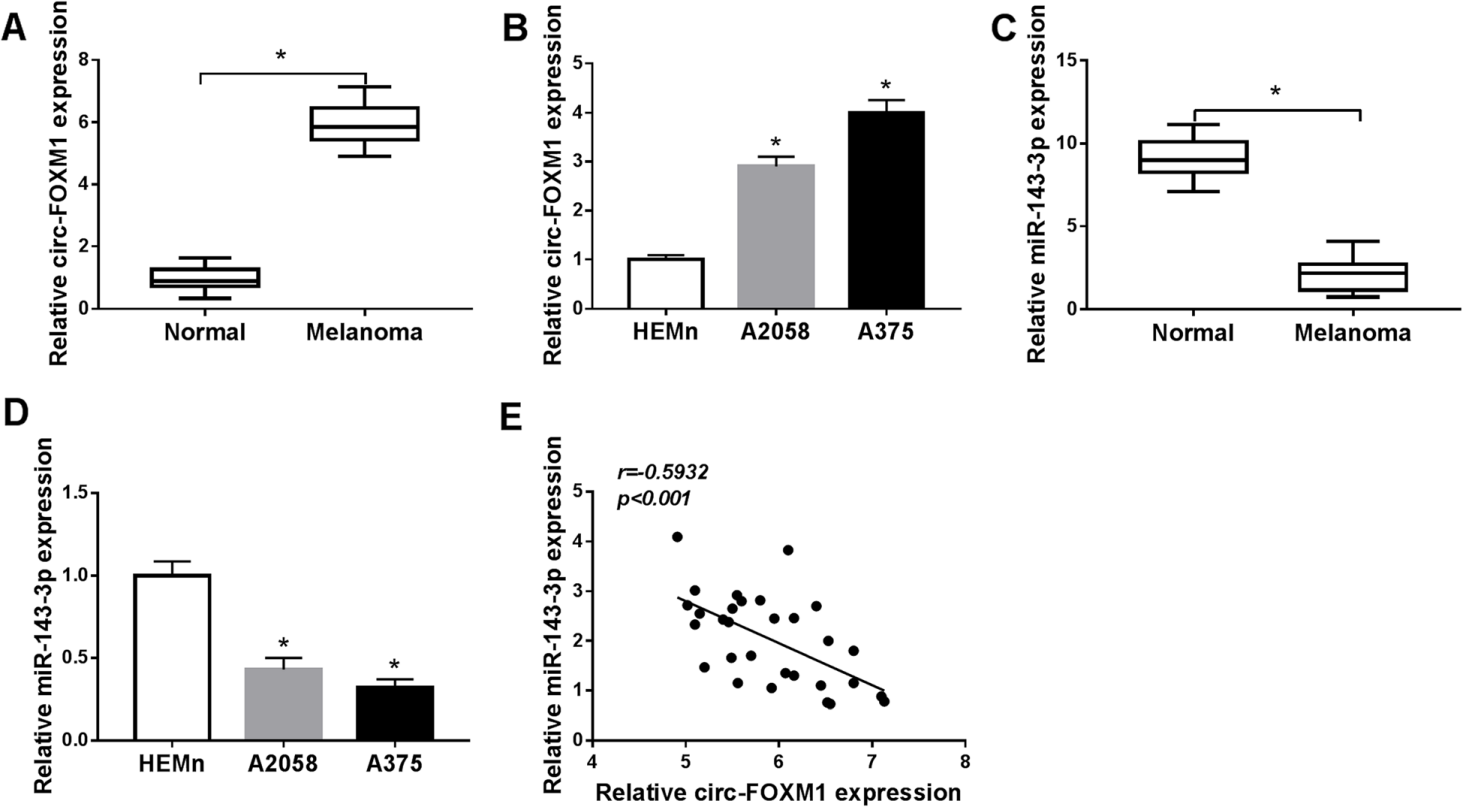
High expression of circ-FOXM1 and low expression of miR-143-3p were observed in melanoma tissues and cells. **a**, **b** The expression level of circ-FOXM1 in melanoma tissues and cells and matched normal tissues and cells was determined by qRT-PCR. **c**, **d** The expression level of miR-143-3p in melanoma tissues and cells and matched normal tissues and cells was determined by qRT-PCR. **e** The correlation between circ-FOXM1 and miR-143-3p was analyzed by Spearman’s correlation coefficient analysis. *P < 0.05

To validate the potential role of circ-FOXM1 in melanoma development, the expression of circ-FOXM1 in 30 melanoma tissues and corresponding normal skin tissues was firstly tested by qRT-PCR. The data showed that circ-FOXM1 expression was markedly raised in melanoma tissues in reference to normal tissues (Fig. 1a). The analysis of circ-FOXM1 in melanoma cells (A2058 and A375) and normal human epidermal melanocytes (HEMn) indicated that circ-FOXM1 was highly expressed in A2058 and A375 cells compared to HEMn cells (Fig. 1b). Subsequently, the expression level of miR-143-3p in melanoma tissues and cells was analyzed. The results of qRT-PCR exhibited that miR-143-3p was conspicuously decreased in melanoma tissues and cells compared to that in normal skin tissues and HEMn cells (Fig. 1c, d). Moreover, there was an inverse correlation between the expression of circ-FOXM1 and miR-143-3p in melanoma tissues, as illustrated by Spearman’s correlation coefficient analysis (Fig. 1e). These results suggested that the dysregulation of circ-FOXM1 and miR-143-3p might play vital roles in melanoma.

### Silencing of circ-FOXM1 inhibited cell proliferation, invasion, and glycolysis and induced apoptosis in melanoma cells

**Fig. 2:**
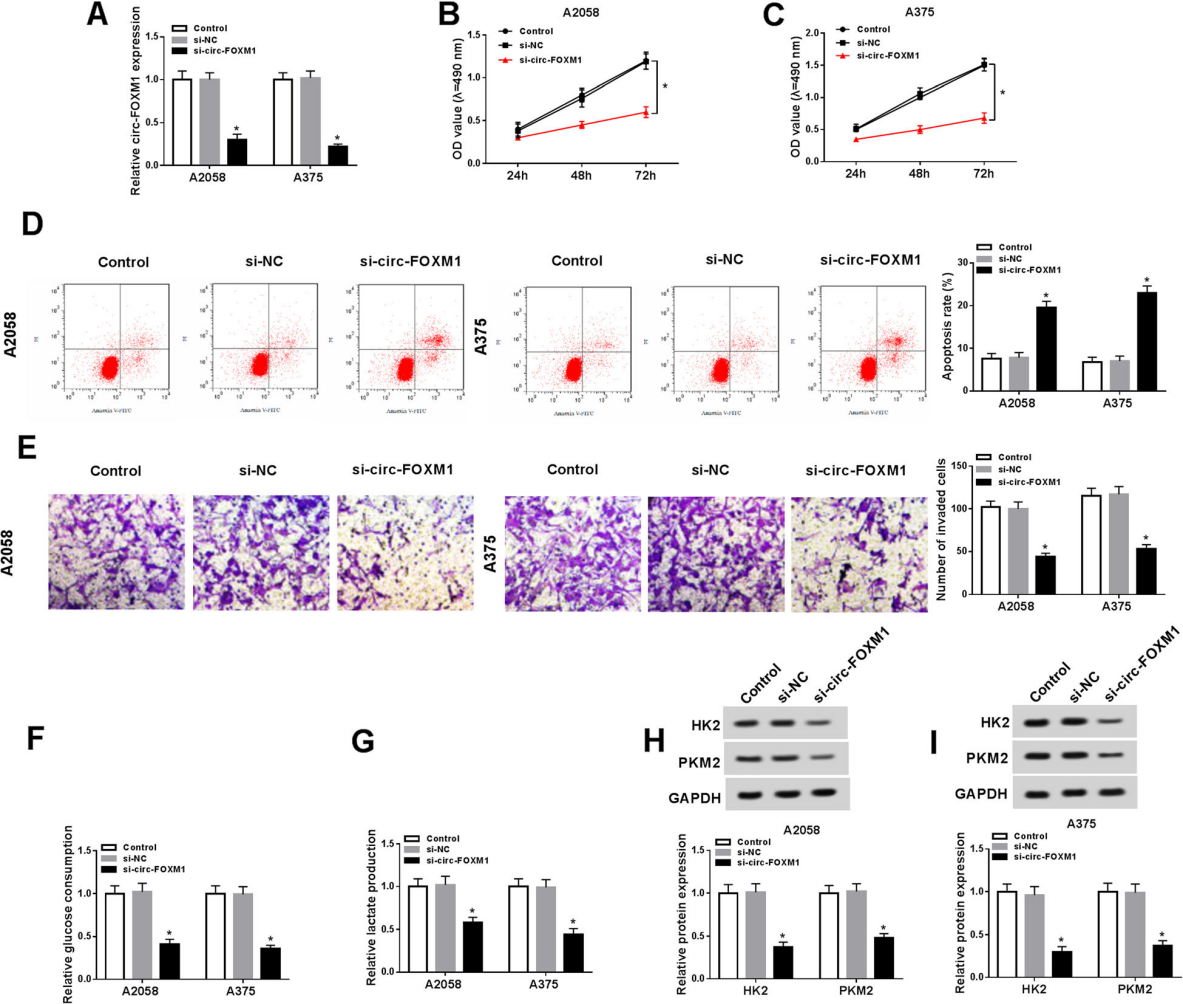
Deficiency of circ-FOXM1 impeded melanoma cell proliferation, invasion, and glycolysis and promoted apoptosis. Si-NC or si-circ-FOXM1 was transfected into A2058 and A375 cells. **a** The expression of circ-FOXM1 in A2058 and A375 cells was measured by qRT-PCR assay. **b**, **c** The proliferation of A2058 and A375 cells was measured through MTT assay. **d** The apoptosis of A2058 and A375 cells was determined via flow cytometry analysis. **e** The invasion of A2058 and A375 cells was evaluated using transwell assay. **f**, **g** The levels of glucose consumption and lactate production were detected by specific kits. **h**, **i** The protein levels of HK2 and PKM2 were measured through western blot assay. *P < 0.05

In order to reveal the exact roles of circ-FOXM1 in melanoma development, si-circ-FOXM1 was transfected into A2058 and A375 cells to downregulate circ-FOXM1 expression. As shown in Fig. 2a, si-circ-FOXM1 transfection led to a remarkable reduction of circ-FOXM1 expression in A2058 and A375 cells. MTT assay proved that cell proliferation was drastically suppressed in A2058 and A375 cells following the deficiency of circ-FOXM1 compared to si-NC group (Fig. 2b, c). The results of flow cytometry analysis exhibited that the apoptosis of A2058 and A375 cells was distinctly induced after circ-FOXM1 knockdown compared to control group (Fig. 2d). The data of transwell assay indicated that cell invasion was markedly inhibited in si-circ-FOXM1 transfected A2058 and A375 cells compared to si-NC transfected cells (Fig. 2e). Furthermore, whether circ-FOXM1 regulated the glycolysis of melanoma cells was explored via detecting the levels of glucose consumption, lactate production, and glycolysis key enzymes (including HK2 and PKM2). The results implied that the levels of glucose consumption, lactate production, HK2, and PKM2 were all repressed in A2058 and A375 cells by circ-FOXM1 downregulation compared to control groups (Fig. 2f–i), indicating that circ-FOXM1 knockdown repressed glycolysis in melanoma cells. Collectively, circ-FOXM1 silencing suppressed melanoma cell progression via regulating cell proliferation, apoptosis, invasion, and glycolysis.

### Circ-FOXM1 regulated cell proliferation, apoptosis, invasion, and glycolysis by targeting miR-143-3p in melanoma cells

**Fig. 3:**
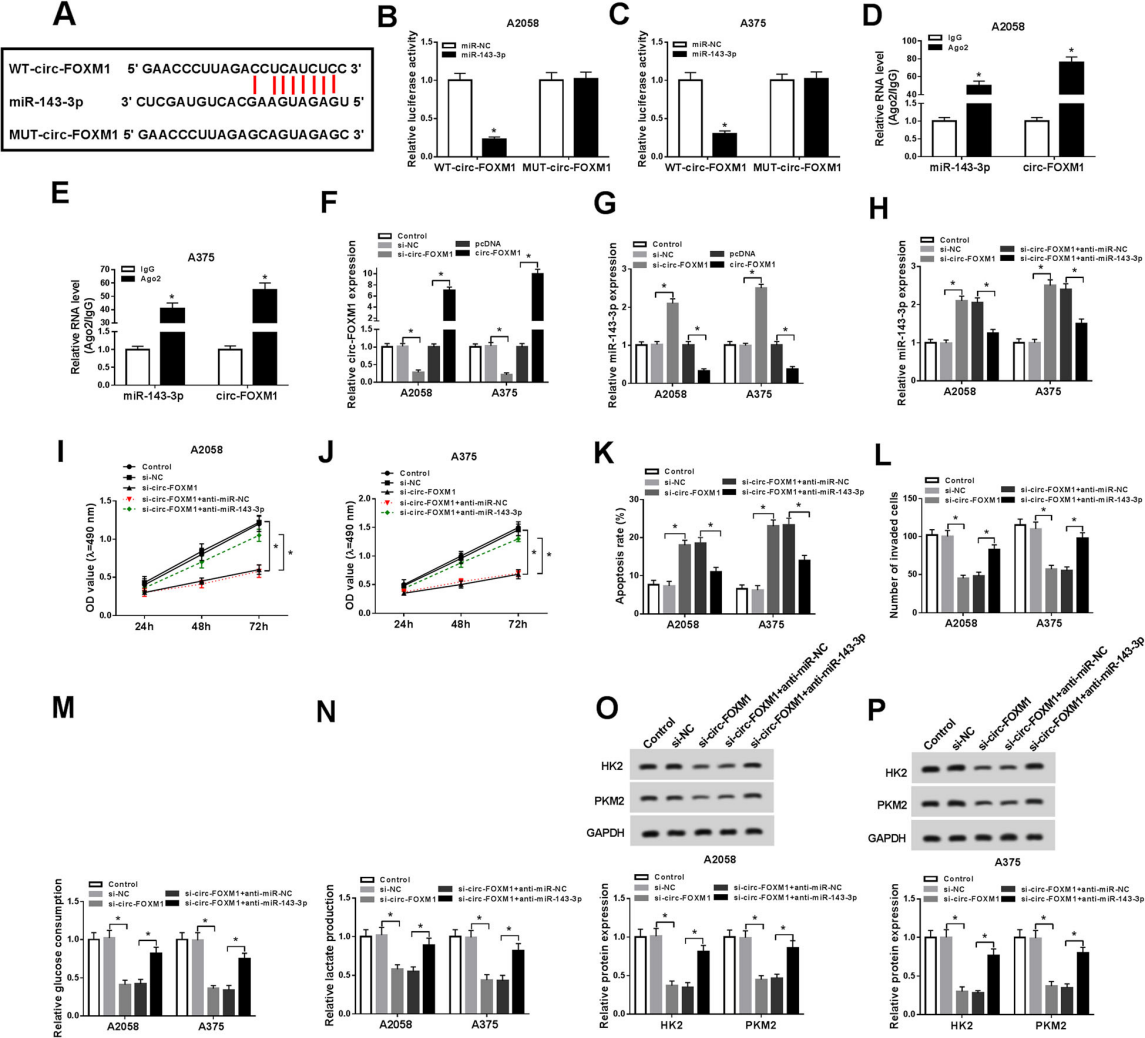
Circ-FOXM1 directly targeted miR-143-3p to regulate melanoma cell proliferation, apoptosis, invasion, and glycolysis. **a** The binding sites between circ-FOXM1 and miR-143-3p were predicted by starBase v2.0. **b**, **c** MiR-NC or miR-143-3p in combination with WT-circ-FOXM1 or MUT-circ-FOXM1 were transfected into A2058 and A375 cells, and then the luciferase activity was determined by dual-luciferase reporter assay. **d**, **e** The levels of miR-143-3p and circ-FOXM1 in Ago2 or IgG immunoprecipitates in A2058 and A375 cells were measured by RIP assay and qRT-PCR assay. **f**, **g** A2058 and A375 cells were untransfected or transfected with si-NC, si-circ-FOXM1, pcDNA, or circ-FOXM1 and then the expression levels of circ-FOXM1 and miR-143-3p were detected by qRT-PCR. **h**–**p** A2058 and A375 cells were assigned to control, si-NC, si-circ-FOXM1, si-circ-FOXM1 + anti-miR-NC, and si-circ-FOXM1 + anti-miR-143-3p groups. **h** The expression of miR-143-3p in A2058 and A375 cells was examined by qRT-PCR. **i**, **j** A2058 and A375 cell proliferation was evaluated by MTT assay. **k** A2058 and A375 cell apoptosis was analyzed by flow cytometry. **l** A2058 and A375 cell invasion was assessed by transwell assay. **m**, **n** The levels of glucose consumption and lactate production in A2058 and A375 cells were determined by relevant kits. **o**, **p** The protein levels of HK2 and PKM2 in A2058 and A375 cells were determined via western blot assay. *P < 0.05

To explore the potential mechanism of circ-FOXM1 in melanoma progression, we searched online website starBase v2.0 and found that circ-FOXM1 contained the complementary sequences of miR-143-3p (Fig. 3a), indicating that miR-143-3p might be a target of circ-FOXM1. Dual-luciferase reporter assay displayed that the luciferase activity in miR-143-3p and WT-circ-FOXM1 co-transfected A2058 and A375 cells was inhibited compared to miR-NC and WT-circ-FOXM1 co-transfected cells, whereas the luciferase activity was not changed in MUT-circ-FOXM1 groups (Fig. 3b, c). RIP assay showed that the levels of miR-143-3p and circ-FOXM1 were distinctly increased in Ago2 immunoprecipitates in A2058 and A375 cells compared to IgG immunoprecipitates (Fig. 3d, e). Moreover, we observed that si-circ-FOXM1 transfection led to a marked decrease of circ-FOXM1 expression and a marked increase of miR-143-3p expression in A2058 and A375 cells, while circ-FOXM1 transfection showed the opposite results (Fig. 3f, g). The above data indicated that circ-FOXM1 negatively regulated miR-143-3p expression via direct interaction.

Subsequently, we divided A2058 and A375 cells into 5 groups (control, si-NC, si-circ-FOXM1, si-circ-FOXM1 + anti-miR-NC, and si-circ-FOXM1 + anti-miR-143-3p) to explore whether circ-FOXM1 could alter melanoma cell progression via targeting miR-143-3p. As exhibited in Fig. 3h, the upregulation of miR-143-3p expression caused by circ-FOXM1 knockdown was effectively overturned following miR-143-3p inhibition in both A2058 and A375 cells. As illustrated by MTT assay, flow cytometry analysis, and transwell assay, the deletion of miR-143-3p reversed the inhibitory effects on cell proliferation and invasion and the promotional effect on cell apoptosis mediated by circ-FOXM1 silencing in A2058 and A375 cells (Fig. 3i–l). In addition, the effect of circ-FOXM1 and miR-143-3p on glycolysis level was explored. The data showed that the levels of glucose consumption, lactate production, HK2, and PKM2 were all reduced in A2058 and A375 cells after circ-FOXM1 knockdown, while miR-143-3p inhibition partly restored the impacts (Fig. 3m–p). To sum up, circ-FOXM1 knockdown suppressed melanoma cell proliferation, invasion, and glycolysis and promoted apoptosis by directly targeting miR-143-3p.

### MiR-143-3p negatively regulated FLOT2 expression via directly targeting in melanoma cells

**Fig. 4:**
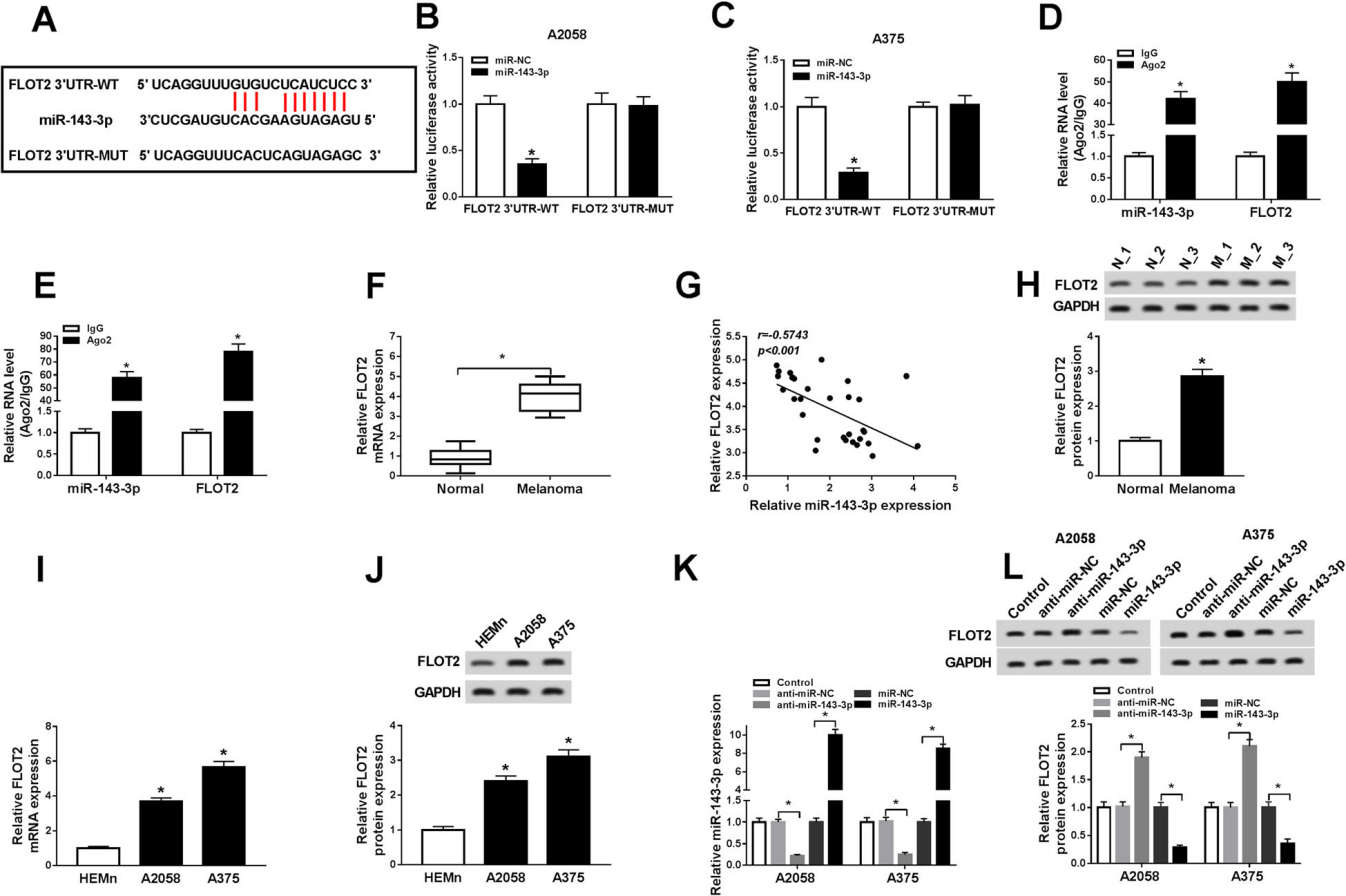
FLOT2 was a direct target gene of miR-143-3p and negatively regulated by miR-143-3p in melanoma cells. **a** The potential targeted gene of miR-143-3p was predicted by starBase v2.0. **b**, **c** The association between miR-143-3p and FLOT2 was investigated by dual-luciferase reporter assay. **d**, **e** The binding efficiency of miR-143-3p and FLOT2 to Ago2 or IgG antibody in A2058 and A375 cells was detected by RIP assay and qRT-PCR assay. **f** The mRNA expression of FLOT2 in melanoma tissues and normal tissues was measured by qRT-PCR. **g** The correlation between miR-143-3p and FLOT2 was analyzed by Spearman’s correlation coefficient analysis. **h** The protein level of FLOT2 in melanoma tissues and normal tissues was determined by western blot assay. **i**, **j** The mRNA and protein levels of FLOT2 in HEMn, A2059, and A375 cells were measured by qRT-PCR assay and western blot assay, respectively. **k**, **l** The levels of miR-143-3p and FLOT2 protein in A2058 and A375 cells transfected with anti-miR-NC, anti-miR-143-3p, miR-NC, or miR-143-3p were determined by qRT-PCR assay and western blot assay, respectively. *P < 0.05

As predicted by starBase v2.0, FLOT2 might be a target gene of miR-143-3p and their potential binding sites were shown in Fig. 4a. To verify it, dual-luciferase reporter assay and RIP assay were performed. The transfection of miR-143-3p and FLOT2 3′UTR-WT caused an obvious suppression in the luciferase activity in A2058 and A375 cells compared to miR-NC and FLOT2 3′UTR-WT co-transfected groups, while no change was observed in FLOT2 3′UTR-MUT groups, as demonstrated by dual-luciferase reporter assay (Fig. 4b, c). The data of RIP assay exhibited that miR-143-3p and FLOT2 were distinctly enhanced in Ago2 immunoprecipitates in A2058 and A375 cells compared to IgG control groups (Fig.4d, e). Next, we determined the mRNA and protein levels of FLOT2 in melanoma tissues and normal skin tissues. As we expected, the mRNA and protein levels of FLOT2 were markedly raised in melanoma tissues relative to normal tissues (Fig. 4f, h). Furthermore, FLOT2 expression was negatively correlated with miR-143-3p expression in melanoma tissues (Fig. 4g). The analysis of FLOT2 mRNA and protein levels in melanoma cells and HEMn cells showed that the mRNA and protein levels of FLOT2 were notably elevated in A2058 and A375 cells compared to HEMn cells (Fig. 4i, j). Besides, we found that anti-miR-143-3p transfection apparently decreased miR-143-3p level but apparently increased FLOT2 protein level in A2058 and A375 cells, whereas miR-143-3p transfection increased miR-143-3p level and decreased FLOT2 protein level (Fig. 4k, l). All these data suggested that miR-143-3p negatively modulated FLOT2 expression by interacting with FLOT2 in melanoma cells.

### FLOT2 overexpression weakened the impacts of miR-143-3p on cell proliferation, apoptosis, invasion, and glycolysis in melanoma cells

**Fig. 5:**
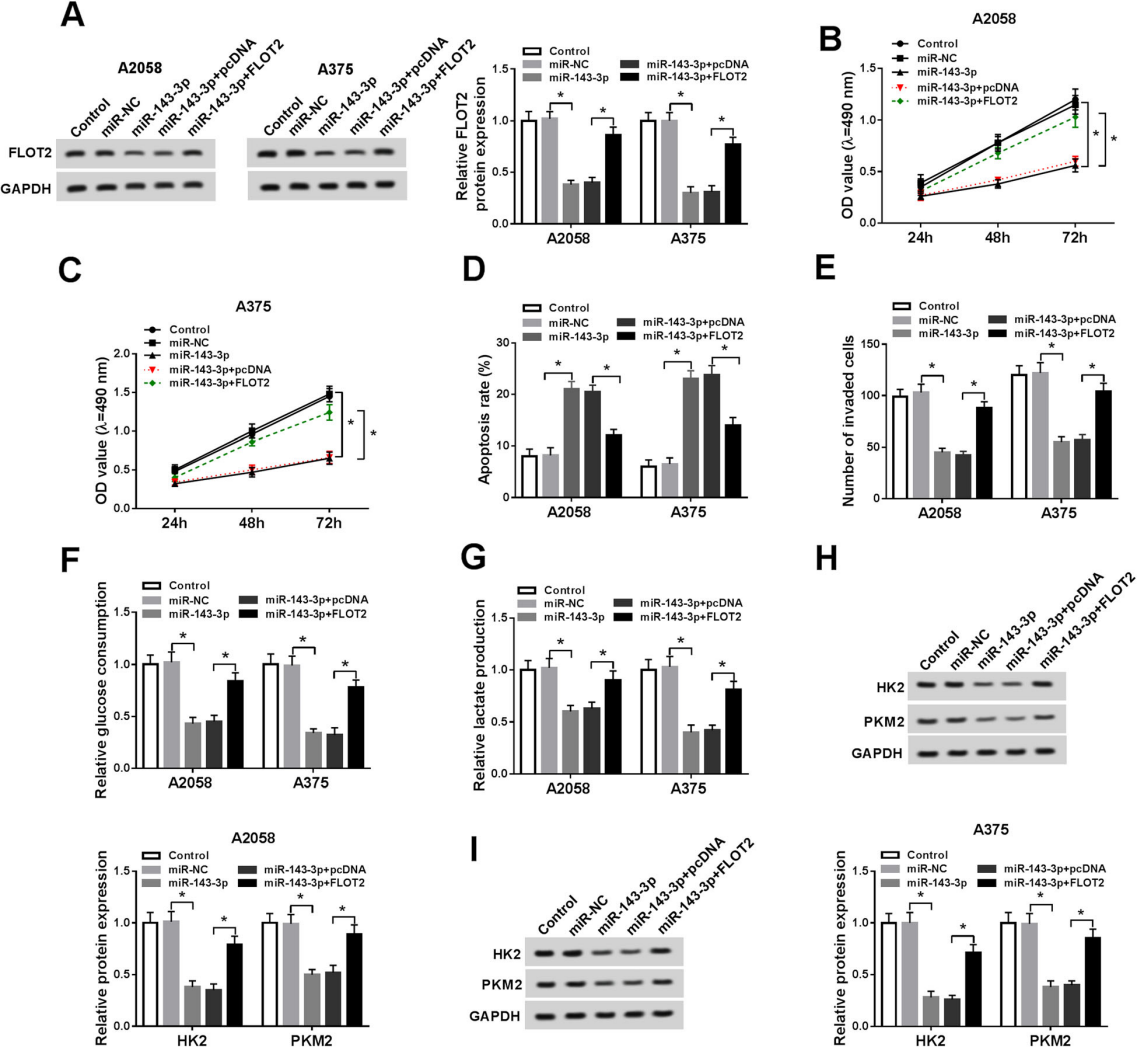
MiR-143-3p suppressed melanoma cell proliferation, invasion, and glycolysis and facilitated apoptosis via binding to FLOT2. A2058 and A375 cells were divided into 5 groups: control, miR-NC, miR-143-3p, miR-143-3p + pcDNA, and miR-143-3p + FLOT2. **a** The protein level of FLOT2 was determined by western blot assay. **b**, **c** The proliferation of A2058 and A375 cells was tested by MTT assay. **d** The apoptosis of A2058 and A375 cells was assessed using flow cytometry analysis. **e** The invasion of A2058 and A375 cells was determined by transwell assay. **f**, **g** The consumption of glucose and the production of lactate were determined using specific kits. **h**, **i** The protein levels of HK2 and PKM2 were measured by western blot assay. *P < 0.05

Based on the above data, we wondered whether miR-143-3p regulated melanoma progression via targeting FLOT2. Thus, we divided A2058 and A375 cells into 5 groups: control, miR-NC, miR-143-3p, miR-143-3p + pcDNA, and miR-143-3p + FLOT2. As presented in Fig. 5a, miR-143-3p overexpression notably repressed FLOT2 protein expression in A2058 and A375 cells, while FLOT2 transfection restored the repression. MTT assay indicated that miR-143-3p remarkably suppressed the proliferation of A2058 and A375 cells, whereas FLOT2 overexpression abolished the impact (Fig. 5b,c). The results of flow cytometry analysis displayed that the apoptosis of A2058 and A375 cells was induced by miR-143-3p, but the elevation of FLOT2 further repressed the effect (Fig. 5d). Cell invasion was conspicuously hampered in A2058 and A375 cells after miR-143 transfection, whereas FLOT2 transfection effectively restored the effect, as demonstrated by transwell assay (Fig. 5e). Moreover, the levels of glucose consumption, lactate production, and glycolysis key enzymes were all reduced by miR-143-3p in A2058 and A375 cells, while FLOT2 overexpression markedly overturned the impacts (Fig. 5f–i). All these outcomes suggested that miR-143-3p could suppress melanoma cell progression by targeting FLOT2.

### Circ-FOXM1 knockdown downregulated FLOT2 expression by targeting miR-143-3p in melanoma cells

**Fig. 6:**
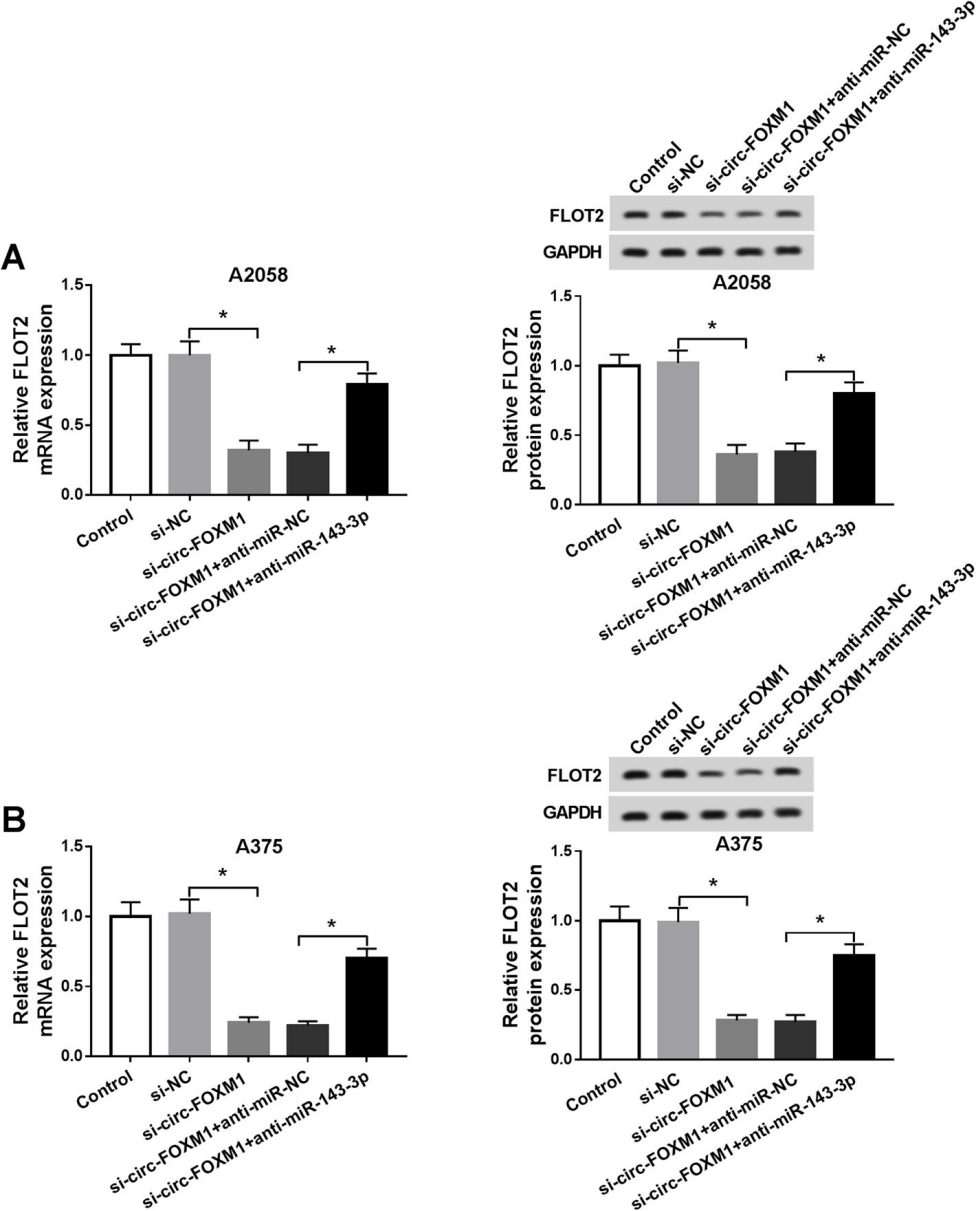
Silencing of circ-FOXM1 decreased FLOT2 expression by sponging miR-143-3p in melanoma cells. **a**, **b** The mRNA and protein levels of FLOT2 in A2058 and A375 cells transfected with si-NC, si-circ-FOXM1, si-circ-FOXM1 + anti-miR-NC, or si-circ-FOXM1 + anti-miR-143-3p and control cells were measured by qRT-PCR assay and western blot assay, respectively. *P < 0.05

We further determined the association among circ-FOXM1, miR-143-3p, and FLOT2. A2058 and A375 cells were transfected with si-NC, si-circ-FOXM1, si-circ-FOXM1 + anti-miR-NC, or si-circ-FOXM1 + anti-miR-143-3p and then the mRNA and protein levels of FLOT2 were detected. The data of qRT-PCR assay and western blot assay exhibited that circ-FOXM1 deficiency resulted in a notable reduction of FLOT2 mRNA and protein expression in A2058 and A375 cells, but miR-143-3p inhibition partially restored the reduction (Fig. 6a, b). Thus, we concluded that circ-FOXM1 could modulate FLOT2 expression through sponging miR-143-3p.

### Deficiency of circ-FOXM1 suppressed tumor growth in vivo

**Fig. 7:**
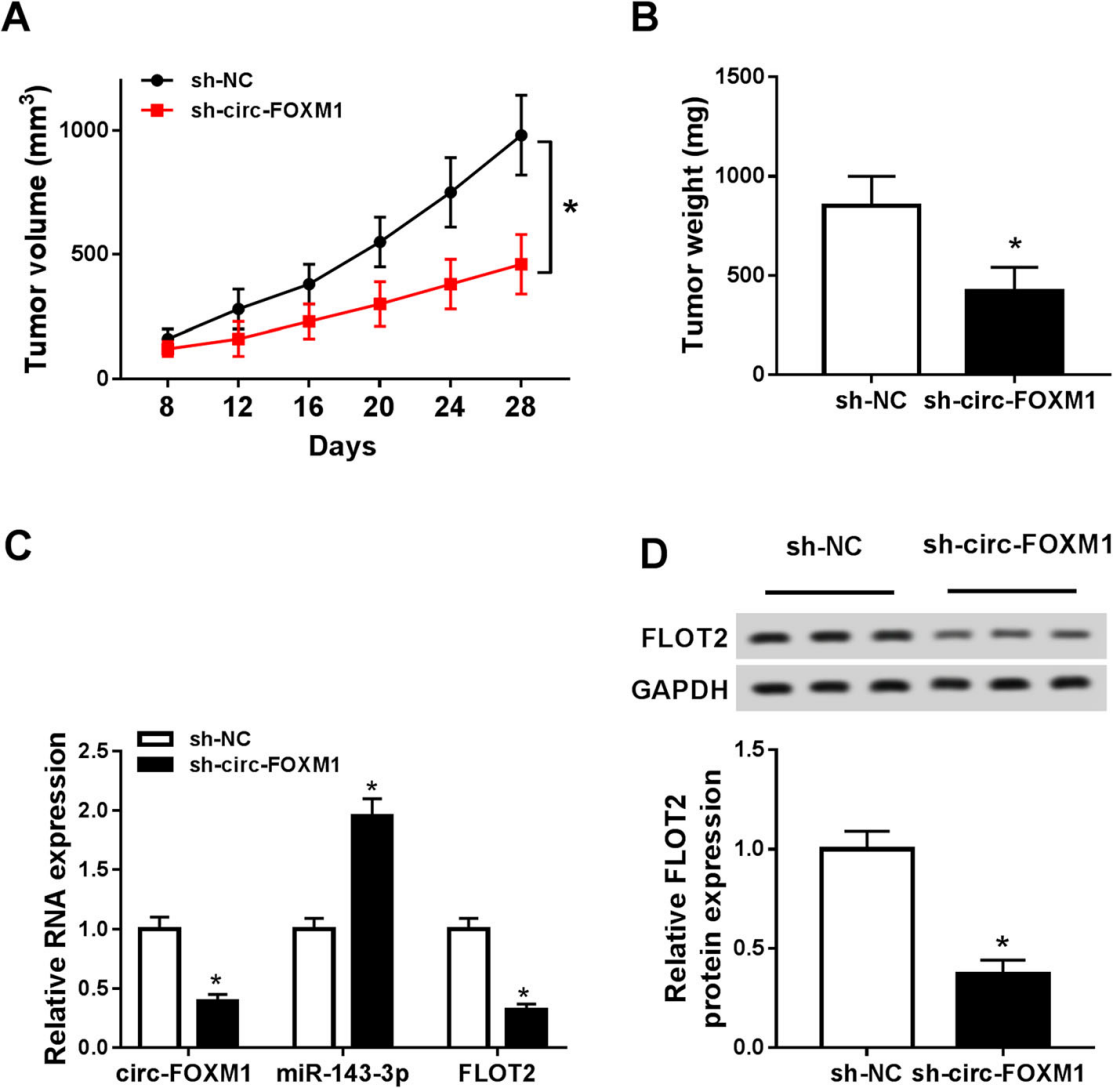
Knockdown of circ-FOXM1 hampered tumor growth in vivo. Sh-circ-FOXM1 or sh-NC transfected A375 cells were injected into the mice. **a** Tumor volume was monitored every 4 days from the 8th day. **b** Tumor weight was measured after 28 days of injection. **c** The levels of circ-FOXM1, miR-143-3p, and FLOT2 mRNA were determined by qRT-PCR. **d** The protein level of FLOT2 was measured by western blot assay. *P < 0.05

To investigate the function of circ-FOXM1 in tumorigenesis in vivo, sh-circ-FOXM1 or sh-NC stably transfected A375 cells were injected into the mice to construct murine xenograft models. As shown in Fig. 7a and b, tumor volume and weight were conspicuously blocked in sh-circ-FOXM1 groups compared to sh-NC control groups. Next, we determined the levels of circ-FOXM1, miR-143-3p, FLOT2 mRNA, and FLOT2 protein in the collected tumor samples. The data showed that the levels of circ-FOXM1, FLOT2 mRNA, and FLOT2 protein were decreased and the level of miR-143-3p was increased in sh-circ-FOXM1 groups relative to sh-NC groups (Fig. 7c, d). Collectively, circ-FOXM1 knockdown blocked tumorigenesis in vivo.

## Discussion

A large number of studies have revealed that circRNAs are closely related to disease occurrence and development and have potential applications in disease diagnostic markers, but their biological functions are still largely unknown [17]. In the paper, we elucidated the effects of circ-FOXM1 in melanoma. We observed that circ-FOXM1 was obviously elevated in melanoma tissues and cells. Moreover, our data exhibited that circ-FOXM1 deficiency repressed cell proliferation, metastasis, and glycolysis and facilitated apoptosis in melanoma by miR-143-3p/FLOT axis.

It has been documented that circRNAs can sponge miRNAs to suppress their functions [18]. Several circRNAs were verified to be associated with melanoma progression. For example, Jin et al. manifested that circMYC was elevated in melanoma and facilitated melanoma cell growth and glycolysis [19]. Luan et al. verified that circ_0084043 was highly expressed in melanoma and facilitated melanoma cell proliferation and motility by directly interacting with miR-153-3p [20]. Bian et al. proved that the circ_0025039 level was raised in melanoma and its silencing hampered melanoma cell growth, colony formation, metastasis, and glycolysis by binding to miR-198 [21]. In line with these data, we revealed that circ-FOXM1 was conspicuously elevated in melanoma tissues and cells. By loss-of-function experiments, we discovered that the downregulation of circ-FOXM1 led to a marked inhibition in melanoma cell proliferation, metastasis, and glycolysis and a remarked promotion in melanoma cell apoptosis. Moreover, circ-FOXM1 deficiency blocked tumorigenesis in vivo. The outcomes illustrated that circ-FOXM1 acted as an oncogene in melanoma. Additionally, miR-143-3p was found to be weakly expressed in melanoma and served as a target of circ-FOXM1. Deletion of miR-143-3p restored the impacts of circ-FOXM1 silencing on cell proliferation, metastasis, and glycolysis in melanoma. All these data unraveled that circ-FOXM1 knockdown decelerated melanoma progression by sponging miR-143-3p.

Previous reports have shown that miR-143-3p was abnormally expressed in various cancers [22, 23]. In melanoma, Panza et al. revealed that miR-143-3p was diminished and miR-143-3p elevation impeded melanoma cell growth and motility and induced apoptosis by binding to COX-2 [14]. Li et al. confirmed that miR-143 was reduced in melanoma and miR-143 upregulation hampered melanoma cell growth and facilitated apoptosis by binding to Syn-1 [24]. Consistently, we observed that overexpression of miR-143-3p hampered cell growth, motility, and glycolysis and facilitated apoptosis in melanoma. Moreover, FLOT2 was confirmed to be a target gene of miR-143-3p. Hazarika et al. reported that FLOT2 elevation promoted cell progression and metastasis in SB2 melanoma cells [15]. Doherty et al. suggested that high level of FLOT2 was related to lymph node metastasis in melanoma [25]. Moreover, Liu et al. disclosed that FLOT2 could be targeted by miR-34a and participated in the suppressive roles of miR-34a in melanoma cell proliferation and motility [16]. Herein, FLOT2 elevation abrogated the influences of miR-143-3p on cell growth, metastasis, and glycolysis in melanoma, indicating that miR-143-3p altered melanoma cell development via interacting with FLOT2.

## Conclusion

In summary, circ-FOXM1 was upregulated in melanoma, and circ-FOXM1 contributed to melanoma cell proliferation, motility, and glycolysis and repressed apoptosis by upregulating FLOT2 via targeting miR-143-3p. Our data provided a novel regulatory network circ-FOXM1/miR-143-3p/FLOT2 axis in melanoma progression and might have a crucial implication for melanoma treatment.

## Data Availability

The datasets used and/or analyzed during the current study are available from the corresponding author on reasonable request.

## Abbreviations

FLOT2: Flotillin 2
HK2: Hexokinase2
PKM2: Pyruvate kinase isozyme type M2
